# The Influence of *Ficus deltoidea* in Preserving Alveolar Bone in Ovariectomized Rats

**DOI:** 10.1155/2020/8862489

**Published:** 2020-12-24

**Authors:** N. I. Omar, B. Baharin, S. F. Lau, N. Ibrahim, N. Mohd, A. Ahmad Fauzi, N. Muhammad, N. M. Fernandez

**Affiliations:** ^1^Unit of Periodontology, Department of Restorative Dentistry, Faculty of Dentistry, Universiti Kebangsaan Malaysia, Kuala Lumpur, Malaysia; ^2^Faculty of Veterinary Medicine, Universiti Putra Malaysia, Seri Kembangan, Selangor Darul Ehsan, Malaysia; ^3^Department of Craniofacial Diagnostics and Biosciences, Faculty of Dentistry, Universiti Kebangsaan Malaysia, Level 17, Preclinical Building, Kuala Lumpur, Malaysia; ^4^Pharmacology Department, Faculty of Medicine, Universiti Kebangsaan Malaysia Medical Centre, Kuala Lumpur, Malaysia

## Abstract

*Ficus deltoidea* has been shown to possess antioxidant properties that could prevent the development of chronic inflammatory bone diseases. In this study, the efficacy of *F. deltoidea* in preventing alveolar bone resorption in osteoporotic rats induced by ovariectomy (OVX) was investigated. Twenty-four female Wistar rats were divided into four groups (*n* = 6) consisting of sham-operated (SO), ovariectomized control (OVXN), ovariectomized treated with estrogen (OVXP), and ovariectomized treated with *F. deltoidea* extract (OVXF). At the beginning of the study, two nonovariectomized, healthy rats were sacrificed to serve as baseline (BL). Treatment of the rats commenced two weeks after ovariectomy—the OVXP rats that served as positive control received Premarin® (64.5 *μ*g/kg body weight), while OVXF rats were given *F. deltoidea* (800 mg/kg body weight); both agents were administered orally for two months. The negative control group of rats (OVXN) and the SO group received deionized water, also administered via oral gavage. At necropsy, morphometric assessment of the interradicular bone of the first molar was carried out using a micro-CT scanner, while quantification of osteoclasts and osteoblasts was performed histologically. The results showed that no statistically significant differences among the groups (*p* > 0.05) for bone morphometric assessment. However, trabecular thickness in the OVXF group was similar to BL, while trabecular separation and alveolar bone loss height were lower than those of the OVXN group. Histologically, the OVXF group demonstrated a significantly lower number of osteoclasts and a higher number of osteoblasts compared with OVXN (*p*=0.008 and *p*=0.019, respectively; *p* < 0.05). In conclusion, *F. deltoidea* has the capacity to prevent alveolar bone loss in ovariectomy-induced osteoporosis rats by potentially preserving trabecular bone microarchitecture and to decrease osteoclast and increase osteoblast cell count.

## 1. Introduction

Periodontal disease is an infection that leads to the inflammation of the supporting tissues surrounding the teeth, with progressive ligament attachment loss and bone deterioration [1]. Osteoporosis, on the other hand, is defined as low bone mineral density (BMD) that increases the risk of fracture and can be detected using a dual-energy X-ray absorptiometry (DEXA) scan [2].

A previous study showed that osteoporosis could accelerate the alveolar bone resorption caused by periodontitis, thereby indicating that there is a possible association between osteoporosis and periodontal disease [3]. Interestingly, both diseases have been shown to increase the production of cytokines that stimulate osteoclast activity [4]. The disruption of homeostasis involving bone remodeling, hormonal balance, and the progression and resolution of inflammation may result in the presentation of these diseases [5]. Postmenopausal osteoporosis in women is closely linked to estrogen deficiency due to the decline in ovarian functions. This condition has been associated with an imbalance between osteoblast and osteoclast activities due to the oxidative stress and overactivity of reactive oxygen species (ROS) [6]. In periodontal disease, ROS may play a role in the direct degradation of connective tissue components and potentially cause modifications to the structures of the connective tissues, thereby resulting in the loss of periodontal tissue function [7].

Recent studies have shown that women who have postmenopausal osteoporosis tend to experience alveolar bone resorption that can lead to tooth loss and subsequently affect their quality of life [3, 5]. At present, the common treatments for postmenopausal osteoporosis such as estrogen replacement therapy, bisphosphonates, and injectable parathyroid hormones are expensive and can cause complicated side effects [8]. For example, long-term hormonal replacement therapy is correlated with an increased risk of cancer in estrogen-target tissues and stroke [9, 10]. In addition, the prolonged use of bisphosphonates has been reported to be associated with an increased risk of atypical femoral fractures [11, 12].

*Ficus deltoidea* or locally known as “mas cotek” is a Malaysian medicinal plant that is traditionally used to promote general health. The plant contains high amounts of phenolic acids and flavonoids that are responsible for scavenging of the reactive oxygen species during oxidative stress [13]. In a previous study using a rabbit long-bone model, it was observed that flavonoids exerted inhibitory effects on bone resorption [14]. Hence, the antioxidant properties of *F. deltoidea* can be exploited pharmaceutically for the prevention and treatment of diseases implicated by oxidative stress and chronic inflammation of the bone such as osteoporosis and periodontitis. However, the beneficial effects of *F. deltoidea* on the alveolar bone have not been scientifically studied.

In this study, 26 female Wistar rats were used to investigate the efficacy of *F. deltoidea* in preventing alveolar bone resorption in osteoporosis. Rats were selected as the animal model in this study due to the similarities of their periodontal anatomy with human molar regions and their ease of handling as compared with other animals such as dogs or nonhuman primates [15]. The rats were ovariectomized to induce osteoporosis and alveolar bone loss. The extract intervention of *F. deltoidea* was given orally, and the alveolar bone loss was measured by three-dimensional morphometric and histological analyses. A comparative analysis of the results was performed with the baseline, sham-operated, negative control, and positive control groups.

## 2. Materials and Methods

### 2.1. Extract Preparation

Powdered leaves of *F. deltoidea* were purchased from HERBagus Sdn Bhd, Malaysia (UKMB40435). The plant extract was prepared by soaking 100 g/L of *F. deltoidea* powdered leaves in 80% ethanol solvent for 3 days continuously. The ethanol solvent was filtered daily using a Whatman filter paper at room temperature [16]. The *F. deltoidea* extract solution was evaporated to dryness under a vacuum using a rotary machine at 40°C until a dark brown residue was formed. The residue was lyophilized at −80°C for seven days using a freeze dryer, and the powdered *F. deltoidea* extract was stored at 4°C until further use.

### 2.2. Animals

Twenty-six female Wistar rats (12 weeks old) weighing approximately 250–300 g each were obtained from the Laboratory Animal Resource Unit, University Kebangsaan Malaysia (UKM). Female Wistar rats were involved in this study to stimulate estrogen deficiency osteoporosis by ovariectomy. The rats (*n* = 26) were further divided into a baseline group (*n* = 2) and four other treatment groups (*n* = 6). Based on the “resource equation” approach, the statistical comparison of the four groups consisting of six rats in each group was performed using a one-way ANOVA [17]. The rats were housed in individually ventilated cages that were maintained at normal room temperature with 12-hour light-dark cycles. The rats were allowed free access to water and food (AIN-93-M semipure diet formulation) approved by the Animal Ethics Committee of University Kebangsaan Malaysia (UKMAEC). All procedures involving the use of the animals were based on the standard operating procedures approved by the UKMAEC with the reference number: FPG/2017/BADIAH/27-SEPT./866-SEPT.-2017-APR.-2019-AR-CAT2.

### 2.3. Experimental Procedures

**Figure 1:**
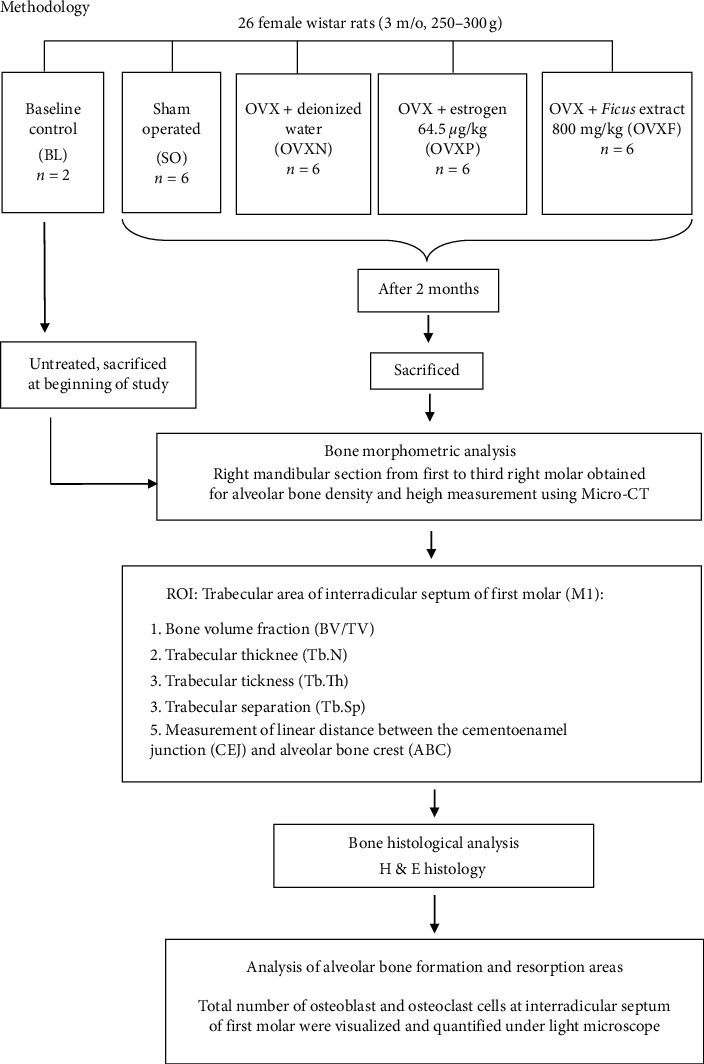
Flowchart diagram of the research methodology involving the stage of intervention, sacrifice, and bone morphometric and histological analysis.

The rats were divided randomly into a baseline group that consisted of two rats and four other groups with six rats (*n* = 6) in each group (Figure 1). The baseline (BL) rats were sacrificed by anesthetic overdose using ketamine (300 mg/kg) in combination with xylazine (30 mg/kg) via intraperitoneal injection at the beginning of the study. The first group consisted of sham-operated (SO) rats, and the remaining 18 rats were ovariectomized (OVX) and further divided into another three groups; nontreated ovariectomized (OVXN), OVX with estrogen (OVXP), and OVX with *F. deltoidea* (OVXF). All treatments were administered daily via oral gavage in the afternoon at the pharmacology laboratory for two months. The direct administration by oral gavage using a rounded-tip “needle” syringe through the diastema (space between incisors and molars) was performed and the intake of the substances was measured accurately.

The treatments were performed two weeks postoperatively to provide sufficient time for wound healing and estrogen hormone levels to decline. The sham-operated and ovariectomized nontreated groups (OVXN) received deionized water, and the ovariectomized estrogen group (OVXP) received conjugated estrogen, Premarin® (64.5 *μ*g/kg/day). The ovariectomized test groups were treated with *F. deltoidea* extract (OVXF). The extracts were dissolved in deionized water and administered orally (10 mL/kg) at a dose of 800 mg/kg/body weight daily. At the end of the study, all the rats were sacrificed by anesthetic overdose using ketamine (300 mg/kg) in combination with xylazine (30 mg/kg) via intraperitoneal injection. Mandibles with intact surrounding tissue from each sacrificed rat were dissected and fixed in a freshly prepared solution containing 10% paraformaldehyde.

### 2.4. Induction of Osteoporosis by Ovariectomy Procedure

The ovariectomized procedure was bilateral ovariectomy, and it was performed on anesthetized rats under surgically sterile conditions via a midline incision using surgical blade No. 11. The rats were anesthetized via intravenous injection at the lateral vein using a mixture of anesthetic solution containing 5% ketamine (0.1 mL per 100 g/body weight) and 2% xylazine (0.01 mL per 100 g/body weight). After the completion of the surgical procedure, the wounds were irrigated, sutured, and covered with an intramuscular antibiotic, Batryl® (0.1 mL per 100 g/body weight), to prevent infection.

### 2.5. Micro-CT Imaging

The right mandibular section from the first to the third molar region of each group was scanned using the Skyscan 1076® Micro-CT scanner system. The X-ray tube was operated at 85 kV and 125 *μ*A using a 0.5 mm Al filter with a resolution of 9.0 *μ*m pixels. Scanning was performed using the following parameters: a 360° rotation around the vertical axis, camera exposure time of 2000 ms, rotation step of 0.88°, and frame averaging of 3. Reconstructions of the micro-CT image slices (18 *μ*m per pixel resolution) were performed with the Skyscan NRecon® software, and the process generated a series of planar transverse gray value images.

### 2.6. Morphometric Analysis of Trabecular Bone

**Figure 2:**
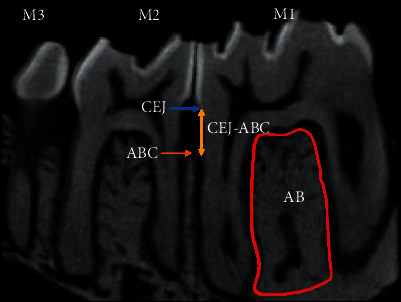
Alveolar bone morphometric analysis area. Analysis of alveolar bone morphometry was done at the interradicular bone of first molar, represented by the red line. Bone volume fraction (BV/TV), trabecular number (Tb.N), trabecular thickness (Tb.Th), and trabecular separation (Tb.Sp) were analyzed. Alveolar bone loss height was measured at the distance between CEJ 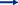 to ABC 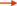. CEJ-ABC represented by 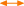 at all four sites of mesiolingual (ML), mesiobuccal (MB), distolingual (DL), and distobuccal (DB) sections of the first molar. AB, alveolar bone; CEJ, cementoenamel junction; ABC, alveolar bone crest; CEJ-ABC, distance of cementoenamel junction to alveolar bone crest; M1, first molar; M2, second molar; M3, third molar.

A three-dimensional (3D) volume of interest (VOI) of the first molar (M1) was standardized and defined based on the following anatomical features: the apex of the mesial root of the M1 (apical limit); the M1 furcation area (coronary limit); mesial of the mesial root of the M1 (anterior limit); and mesial of the mesial root of the second molar (posterior limit) (Figure 2). Bone volume fraction (BV/TV), trabecular number (Tb.N), trabecular thickness (Tb.Th), and trabecular separation (Tb.Sp) were calculated using standard methods at the interradicular septum of M1, extending from the furcation roof to the mesial and distal root apices. The linear distance between the cementoenamel junction (CEJ) and alveolar bone crest (ABC) was measured to reflect the decreased volume of alveolar crest height (ACH). The decreased ACH was obtained by averaging the CEJ-ABC distances measured at the mesiolingual (ML), mesiobuccal (MB), distolingual (DL), and distobuccal (DB) sections of M1.

### 2.7. Hematoxylin and Eosin (H&E) Tissue Preparation

The mandible specimens were decalcified in 10% of formic acid solution for ten days at room temperature. The specimens were washed under running water and processed by an automated tissue processor (Leica Model TP1020®) for 16 hours. The tissue block was then embedded in paraffin wax for sectioning using the Paraffin Embedding System Cool Unit (Leica Model TBS88®). The paraffin block of the specimen was kept in a freezer at −20°C for several hours.

For the preparation of histology slides, the paraffin block was sliced using a rotary microtome (Leica Model RM2136®). Serial mesiodistal sections (4 *μ*m) parallel to the long axis of M1 were sliced to obtain the desired tissue orientation on the slide [18]. Three slides were prepared at the M1 interradicular bone region, consisting of the buccal section, middle section, and lingual section of the bone with a 20-slice interval (20 × 4 *μ*m), equivalent to 80 *μ*m for each section [19]. The tissue sections were examined under a light microscope (Olympus Model CH30®) to confirm that the region of interest required for the analysis was adequate and properly presented. Hematoxylin and eosin (H&E) staining was performed for the sectioned tissues that were confirmed by microscopy.

### 2.8. Quantification of Osteoclasts and Osteoblasts

**Figure 3:**
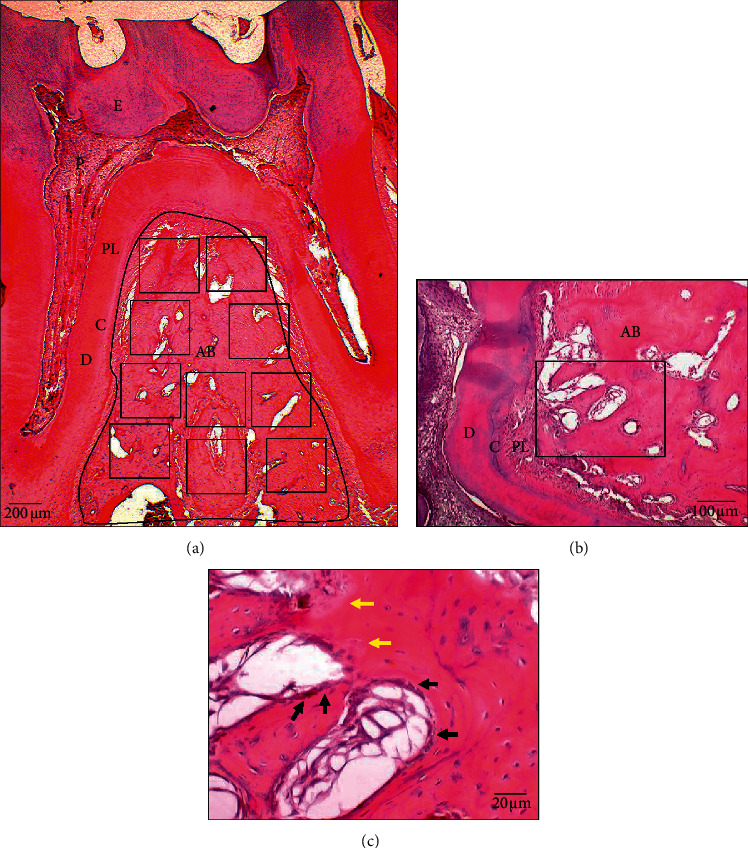
Alveolar bone histological analysis area. (a) The cell number of osteoclasts and osteoblasts cell number were counted at bone remodeling areas 10 hpf (high-power fields) at 4x magnification within the interradicular bone of first molar, represented by the black line. AB, alveolar bone; E, enamel; D, dentine; P, pulp; C, cementum; PL, periodontal ligament. (b) Example of one of the field areas for histological analysis (represented by the black line) at 10x magnification. (c) Quantitation of osteoblasts and osteoclasts under 40x magnification. The mononuclear cell of osteoblasts was lined up the external surface of bone trabeculae and the surface of Haversian canals (represented by 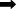). Large multinucleated cells of osteoclasts were located in small resorptive excavations (represented by 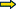).

The differentiation of osteoblasts and osteoclasts in H&E-stained slides as represented by the buccal, middle, and lingual sections of the interradicular bone for each group was visualized under a light microscope (Olympus Model JC30®). At the interradicular bone of M1, ten fields of bone remodeling areas depicting the outer trabecular and intratrabecular areas were observed under 4x magnification and selected for the cell count area [20] (Figure 3(a)). The quantitation of osteoblasts and osteoclasts were determined by morphologically quantifying the number of cells at a magnification of 40x [21] (Figure 3(c)). Osteoblasts were counted as mononuclear cells arranged at the external surface of the bone trabeculae and surface of Haversian canals. Large cells consisting of two to fifteen nuclei in small resorptive excavations (Howship's lacunae) on the bone surface were counted as multinuclear osteoclasts [21]. The number of osteoclasts and osteoblasts for each sample was recorded as the mean number of osteoblasts and osteoclasts counted in the slides of all the three sections mentioned above.

### 2.9. Reproducibility and Reliability

Alveolar bone morphometric and linear measurements of CEJ-ABC were analyzed by a single-blind expert examiner in animal diagnostic imaging (LSF). Intraexaminer reproducibility was carried out as a single-blind remeasurement of ten samples randomly given to the examiner by the researcher. The reproducibility of the first measurement and random remeasurements of the ten samples was analyzed using intraclass correlation coefficient (ICC) statistics (ICC = 0.99).

For bone histological analysis, the osteoblasts and osteoclasts were counted by two single-blind examiners who were a trained examiner (N.I.O.) and an oral pathologist (N.I.), respectively. The interexaminer reliability between the two examiners was performed by counting the osteoclasts and osteoblasts from three random samples consisting of nine slides that were selected by the lab assistant. Both examiners took turns in the counting process of the selected slides. The reliability of the number of osteoclasts and osteoblasts from each slide counted by the examiners was statistically analyzed as mentioned previously (ICC = 0.96).

### 2.10. Statistical Analyses

All the data analyses were performed using the IBM SPSS® Data Editor, version 24.0 (IBM, Armonk, NY, USA), and the values were expressed as mean ± standard deviation (SD). The Kolmogorov–Smirnov and Shapiro–Wilk tests were used to determine whether the two data sets differed significantly. A statistical model of a one-way analysis of variance (ANOVA) was used to analyze the mean value differences between the groups for the normally distributed data, while the Kruskal–Wallis test was used to analyze the nonparametric data. The Tukey post hoc test was used in conjunction with ANOVA and Kruskal–Wallis tests to determine if the mean values were significantly different among the groups. A statistical significance at *p* < 0.05 was applied for the results obtained in this study.

## 3. Results

### 3.1. Alveolar Bone Loss Height Measurement

**Figure 4:**
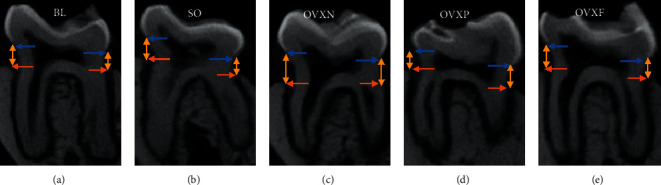
Coronal sections of the alveolar bone showing the height of the bone loss from the CEJ (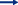) to ABC (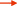) of M1 (CEJ-ABC is represented by 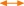) of all groups. B, buccal; L, lingual.

**Figure 5:**
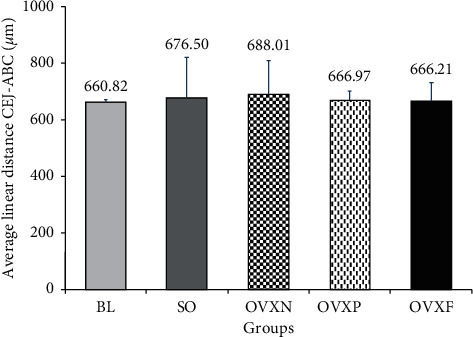
The mean values and standard deviation of the average linear distance of CEJ-ABC for all the groups.

The two-dimensional (2D) micro-computed tomography (micro-CT) image of the coronal section for the M1 alveolar bone loss height measurement revealed that there was no statistically significant differences among the groups (*p* > 0.05). Nevertheless, reduced alveolar bone loss was observed in the OVXF group compared with the OVXN group (*p*=0.995). It was revealed that an increase in bone loss for the OVXN group was related to the other groups (Figure 4). In addition, minimal changes were observed in the mean CEJ-ABC distance among the ovariectomized rats in the OVXN, OVXP, and OVXF groups (Figure 5). As compared with BL, the OVXN group had the highest alveolar bone loss followed by the SO group. On the other hand, the OVXF group demonstrated the lowest alveolar bone loss. There was a similar average CEJ-ABC distance observed between OVXF and OVXP, with both groups displaying delicate bone loss of approximately 6 µm as compared with BL.

### 3.2. Alveolar Bone Morphometric Analyses

**Figure 6:**
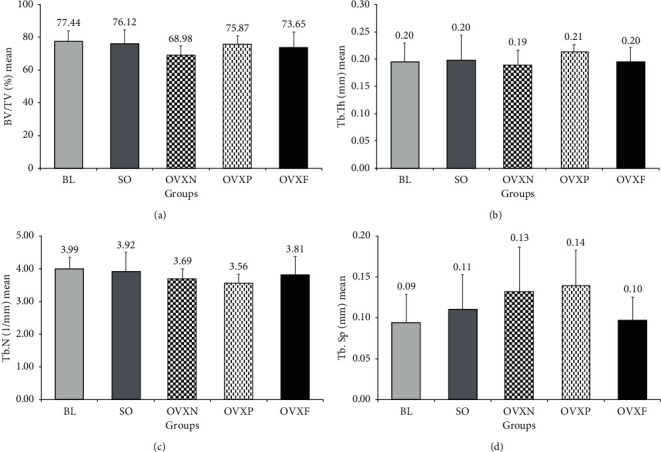
Parameters obtained based on the micro-CT measurements. Mean and standard deviation of (a) bone volume trabecular (BV/TV), (b) trabecular bone thickness (Tb.Th), (c) trabecular bone number (Tb.N), and (d) trabecular bone separation (Tb.Sp) among groups.

**Figure 7:**
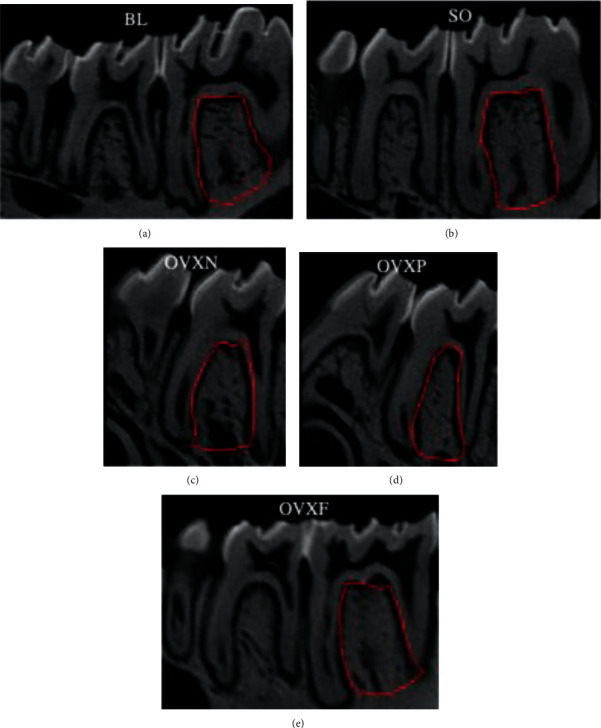
The sagittal section of the interradicular bone of the molar tooth (represented by the red lines).

In the bone morphometric analyses performed in this study, there were no statistically significant differences detected among the groups (*p* > 0.05) for all the parameters evaluated. The OVXN group showed an evidence of alveolar bone loss as seen in the decrease of BV/TV, Tb.N, and Tb.Th, as well as an increase in Tb.Sp (Figure 6). In the 2D micro-CT scan image of the sagittal section of the interradicular bone of M1, the porosity of the interradicular bone in the OVXN group was closely associated with a larger Tb.Sp as compared with BL and other groups (Figure 7). The interradicular bone loss associated with Tb.Sp was quite apparent at the molar apical root region. The interradicular bone morphometry in the SO, OVXP, and OVXF groups appeared to be less porous than that in BL.

In the bone volume fraction (BV/TV) analysis, all ovariectomized groups presented with a minimal reduction of bone volume fraction compared with BL. The same finding was also observed in SO group. It was found that the OVXF group had higher bone volume fraction than the OVXN group, which had the lowest bone volume among the groups (*p* − 0.510). The OVXF group also showed a slight reduction of bone volume compared with the OVXP group and BL (Figure 6(a)).

The mean trabecular thickness (Tb.Th) result has also demonstrated the minimal changes in reduction of the thickness of trabecular bone following two months of ovariectomy in ovariectomized groups. The OVXF group showed a maintained trabecular thickness similar to the SO group and healthy rats in BL. The OVXP group indicated the highest trabecular thickness compared with the other groups. Conversely, the OVXN group had the lowest thickness of trabecular bone (Figure 6(b)).

In the mean trabecular number (Tb.N) analysis, it was found that all ovariectomized groups had experienced reduced trabecular number after ovariectomy when compared with the BL and SO group. The OVXF group revealed higher trabecular number compared with the OVXN group (*p*=0.989). However, an unexpected reduction in Tb.N was found in the OVXP group (Figure 6(c)).

In the mean trabecular separation (Tb.Sp) analysis, the OVXN group indicated a remarkable increment of trabecular separation compared with BL, SO, and OVXF groups. Meanwhile, the OVXF group presented with the lowest trabecular separation compared with SO and two OVX control groups (OVXN and OVXP) (*p*=0.978, *p*=0.593, and *p*=0.416, respectively). Nonetheless, a surprisingly high value of Tb.Sp was found in the group treated with estrogen (OVXP) following osteoporosis (Figure 6(d)).

### 3.3. Histological Analysis

**Figure 8:**
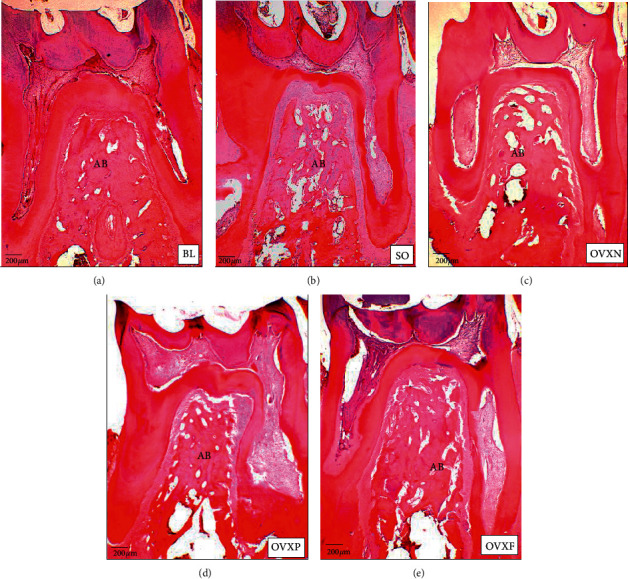
H&E-stained histology images of all groups at the interradicular septum of the first molar after two months of intervention period at 4x magnification. Severe alveolar bone resorption was observed in the OVXN group compared with BL and other groups. AB, alveolar bone; BL, baseline SO, sham-operated; OVXN, ovariectomized; OVXP, ovariectomized + estrogen; OVXF, ovariectomized + *F. deltoidea*.

The H&E-stained histology image of the interradicular septum of the M1 region observed under a light microscope at 4x magnification revealed a prominent osteoporotic trabecular bone in the OVXN group as compared with BL and other groups (Figure 8).

**Figure 9:**
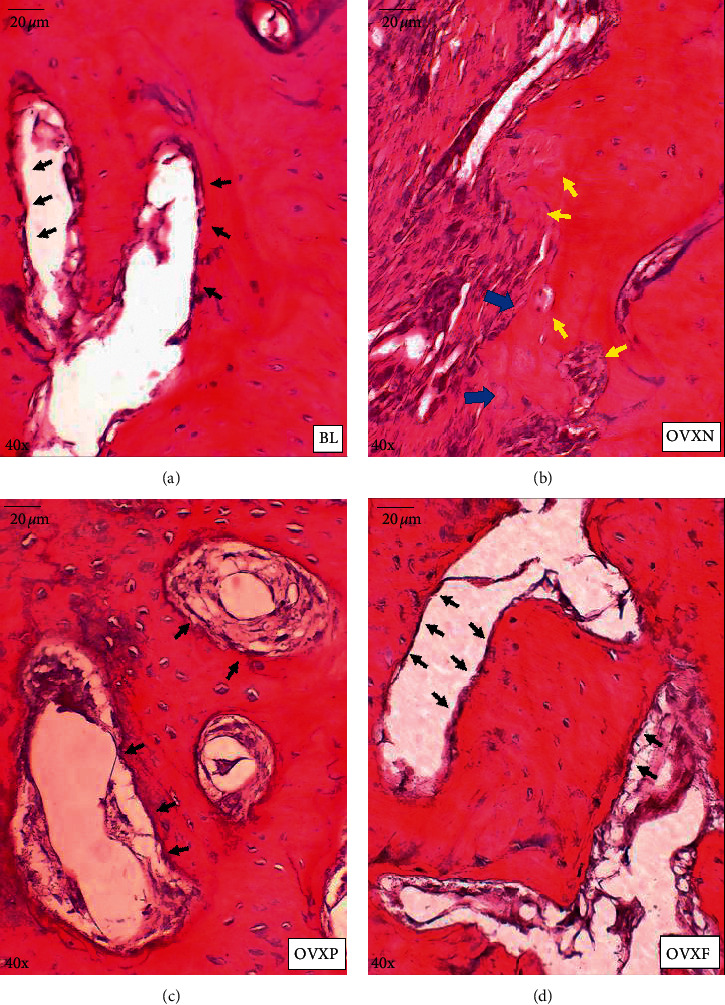
Interradicular septum of the M1 region observed under a light microscope at 40x magnification of (a) baseline rats, (b) OVXN group, (c) OVXP group, and (d) OVXF group. Osteoblasts represented by 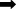; osteoclasts represented by 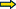; and alveolar bone resorption represented by 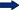.

At 40x magnification, the bone loss was also apparent at the intratrabecular area as opposed to the endosteal surface of the trabecular bone. In the baseline rats (BL), a minimal bone resorption area was observed to be associated with the presence of a small number of osteoclasts and the average number of osteoblasts along the surface of Haversian canals at the intratrabecular area (Figure 9(a)). In the OVXN group, a higher number of bone resorption areas were detected, especially within the intratrabecular region (Figure 9(b)). There were also a significant number of multinucleated osteoclasts and a small number of osteoblasts at the irregular and eroded bone surface areas. In the OVXP group, a small number of osteoclasts with fewer areas of alveolar bone resorption were detected (Figure 9(c)). Additionally, the number of osteoblasts detected was relatively smaller as compared with the BL, SO, and OVXF groups. In the OVXF group, a lower number of osteoclasts and a higher number of osteoblasts were identified (Figure 9(d)). The presence of single-cell osteoblasts arranged along the external surface of the trabecular bone was largely associated with lower bone resorption and increased bone formation areas.

### 3.4. Number of Osteoclasts and Osteoblasts

**Figure 10:**
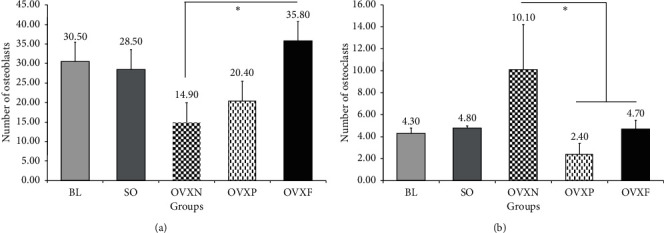
The mean values and standard deviation of the number of osteoclasts and osteoblasts in all the groups. ^*∗*^A significant difference at *p* < 0.05.

The untreated osteoporotic rats in the OVXN group had the highest number of osteoclasts among all the groups investigated in this study (Figure 10(a)). A slight difference in the number of osteoclasts was detected in the OVXF group as compared with the BL and SO groups. Among the ovariectomized groups, the OVXF group had a significantly lower number of osteoclasts as compared with the OVXN group. The OVXP group also demonstrated a significantly smaller number of osteoclasts as compared with the OVXN group. The results were shown to be statistically significant based on their *p* values obtained for OVXF and OVXP groups (*p*=0.008 and *p* < 0.001, respectively; *p* < 0.05).

Nevertheless, the number of osteoblasts in the OVXF group was found to be higher than the BL, SO, and OVXP groups (Figure 10(b)). Among the ovariectomized groups, both OVXF and OVXP displayed a higher number of osteoblasts as compared with the OVXN group. The OVXF group had a significantly higher number of osteoblasts as compared with the OVXN group (*p*=0.019; *p* < 0.05).

## 4. Discussion

### 4.1. Effects of *F. deltoidea* on Alveolar Bone Crest Height

After a two-month intervention period following osteoporosis-induced ovariectomy in rats, the differences in alveolar bone loss height among all the groups as compared with the baseline were not profound, ranging between 6 and 28 *μ*m. Despite no statistically significant difference between the *F. deltoidea* group and the ovariectomized rats, it was shown that the group treated with *F. deltoidea* had less reduction in alveolar crest height than the nontreated ovariectomized group in average and the result was closer to the baseline group.

The alveolar bone processes are covered by a region of compact cortical bone. The nonremarkable changes of alveolar bone loss height among the groups in this study had also been reported in previous analysis of alveolar bone under micro-CT scan following osteoporosis-induced ovariectomy [22]. After sixty days of ovariectomy, a minimal change in the thickness and resorption site of cortical alveolar bone proper compared with the trabecular bone at the interradicular area was found. It was also concluded that the structure of compact cortical bone was the cause for the minimal changes of alveolar bone proper compared to the trabecular bone at the interradicular septum [22]. Recently, two studies have evaluated the effects of ovariectomy on the trabecular bone microarchitecture and cortical bone morphology in the rat mandible using a micro-CT scan morphometric analysis [23, 24]. The authors observed the occurrence of osteoporosis in the trabecular bone microarchitecture of rat mandibles following ovariectomy, although there was no effect observed on the cortical bone morphology [23]. It was also proposed that the alveolar bone loss in the ovariectomized rats was predominantly adjacent to the trabecular bone marrow, suggesting bone loss in postmenopausal osteoporosis occurs mainly at the endosteal surface in the mandible rather than the compact cortical bone surface [24].

Another possible reason accounting for the minimal changes observed for alveolar bone crest height in this present study was due to the absence of periodontitis induction by the ligation method to enhance alveolar bone loss. In a study of the effects of estrogen deficiency on the rat's alveolar bone with experimental periodontitis [25], the outcomes of ligature, ovariectomy, and ovariectomy combined with ligature method to induce osteoporosis on the maxillary alveolar bone were compared. It was concluded that ovariectomy alone did not reduce the alveolar crest height, but more severe reduction found when ovariectomy combined with ligature or ligature alone. The study had also suggested that low alveolar bone mineral density and impaired alveolar bone structure may lead to increased bone resorption of the alveolar crest when periodontitis occurs concurrently with ovariectomy [25]. Hence, it can be suggested that the ovariectomy alone in this study was not able to induce a significant amount of cortical bone loss at the alveolar crest. Therefore, it is thought that the severe reduction in alveolar bone crest height can be induced through the combination of ovariectomy with ligature or ligature alone to evaluate the effect of *F. deltoidea* in preserving the alveolar bone height.

### 4.2. Effects of *F. deltoidea* on Alveolar Bone Microarchitecture

Based on the bone morphometric analyses performed in this study, it was shown that the microarchitecture changes of the interradicular septum following two months ovariectomy in ovariectomized groups was not significantly reduced. However, in the *F. deltoidea* group, it was demonstrated that the trabecular bone thickness was maintained similar to the trabecular bone in the baseline healthy rats. Furthermore, compared with the nontreated ovariectomized rats, the *F. deltoidea* group experienced less bone loss with reduced trabecular separation, and higher bone volume fraction and trabecular number.

The parametric values of the trabecular bone microarchitecture in the femoral necks have been shown to have high correlation with those of the trabecular bone microarchitecture in the mandibles [23]. In our earlier experiment, we observed that the effects of *F. deltoidea* on the trabecular bone microarchitecture of the rats' femora were more remarkable following two months of ovariectomy procedure. In this preliminary study, the herbal extract prevented trabecular bone loss, producing significantly higher BV/TV, Tb.Th, and Tb.*N* values compared with those of OVXN rats. The femoral Tb.Sp of the rats treated with *F. deltoidea* extract was also significantly lower than that of the negative control group. Another significant observation in the rats' long bones is that the OVXF rats displayed similar microarchitecture properties to those of ovariectomized rats treated with estrogen. This is different from the findings in the current study—the estrogen-treated group unexpectedly displayed lower Tb.N and higher Tb.Sp than all other groups. It is found that estrogen treatment could preserve alveolar bone mass in postmenopausal osteoporosis models by reducing osteoclastogenesis and stabilizing bone turnover [26].

We postulate that the lack of significant findings in the current study could either be contributed by the small sample size or the short study duration to produce osteopenia in the mandibular bones. A recent systematic review had stated a significant relationship in mandibular microarchitecture changes and ovariectomy-induced osteoporosis in a rat model [27]. However, heterogeneity was reported where the differences in the region of interest (ROI) and postovariectomy duration had an effect on bone microstructure changes [27]. The well-characterized loss of bone mineral and structure of the long bones and lumbar spine occurs concurrently with a loss of tooth-supporting alveolar bone in the mandible of ovariectomized rats [26]. In the present study, the M1 interradicular septum was evaluated as the ROI in the mandible due to its well-characterized site and distinctive response to estrogen depletion [26]. After two months following the ovariectomy, alveolar bone resorption was observed in rats similarly as reported by earlier studies [22, 28]. Nonetheless, the pattern of deterioration in the alveolar bone microarchitecture in the ovariectomized rats was not consistently affected in all bone morphometric parameters measured. It was found that the OVXN group showed the least changes in trabecular number (Tb.N). This result implies that Tb.N is an ambiguous parameter for detecting bone microstructure changes in the mandible. The Tb.N implicates average trabecular number, which requires a long-term observation until the number of trabecular changes [27]. This particular finding of bone loss in bone morphometric parameters of ovariectomized rats had also been reported in a previous study [29]. After 51 days of ovariectomy surgery, it was reported that ovariectomized mice had lower mandibular trabecular thickness than control, although alveolar bone volume fraction and trabecular separation and number were not significantly affected. Furthermore, in the present study, there was no additional method such as ligation to induce more alveolar bone loss. A study that evaluated changes of bone morphometrics after ninety days in ovariectomized rats with and without ligature had stated that without ligature, the morphometric changes might be minimal [30].

Two recent systematic reviews have mentioned that longer postovariectomy periods were associated with greater effects on bone structural changes in the mandible, and a sufficient ovariectomy duration should be considered as part of the design of studies using ovariectomized rats. However, the duration of postovariectomy required to induce significant bone structural changes was inconclusive [26, 27]. A review study has recommended increasing the duration since ovariectomy up to twelve weeks to observe significant changes in alveolar bone structure [26]. Therefore, to improve this study results, a longer experimental duration is required to observe more trabecular bone loss in the osteoporotic rats after ovariectomy procedure to detect a better finding of *F. deltoidea* effects. Moreover, the combination of ovariectomy and ligation plaque-induced procedures is highly recommended to obtain significant changes in bone morphometric parameters. Additional studies using immunohistochemistry (IHC) analysis should also be performed to validate the presence of the bone resorption marker receptor activator of nuclear factor-kappa-Β ligand (RANKL) and the bone formation marker osteoprotegerin (OPG), to further substantiate the effects of *F. deltoidea* on alveolar bone resorption and expression of formation markers in osteoporotic rats.

Despite the nonsignificant findings in the effect of *F. deltoidea* on alveolar bone microstructure observed in this study, it was exhibited that the bone morphometric parameters in the rats treated with *F. deltoidea* was always more normal in relation to the baseline when compared with the untreated ovariectomized rats. Furthermore, the trabecular thickness was similarly preserved as control healthy rats following two months ovariectomy. It can be suggested that the administration of 800 mg/kg dosage of *F. deltoidea* in the OVXF group has potential to maintain the bone thickness in the mandible and to some extent thus provide protection against the ovariectomy-induced resorption of the trabecular bone structure. This mechanism can be attributed to the activity of *F. deltoidea* in stimulating bone formation and suppressing bone resorption via anti-inflammatory pathways. It was also previously suggested that *F. deltoidea* extract has potent inflammation inhibitory properties that significantly downregulate the expression of bone inflammatory cytokines such as NF-*κβ*, TNF-*α*, and IL-6 to normal levels, as opposed to their excessive increase in osteoporosis [31].

### 4.3. Effects of *F. deltoidea* on the Number of Osteoclasts and Osteoblasts

In comparison among the ovariectomized groups, the ovariectomized with *F. deltoidea* (OVXF) group had revealed a significant effect in reducing the number of osteoclasts and increasing the number of osteoblasts compared with the ovariectomized negative control (OVXN) group with statistically significant difference *p*=0.008 and *p*=0.019(*p* < 0.05), respectively.

The presence of fewer osteoclasts in the OVXF group is likely due to its anti-inflammatory effects that reduce bone resorption by downregulating the proinflammatory mRNA expressions of NF-*κβ*, TNF-*α*, and IL-6, which may lead to the reduction in osteoclast differentiation [24]. In addition, the flavonoid content in *F. deltoidea* possesses potent antioxidant properties, reduces oxidative stress, and eliminates the reactive species [13]. Hence, it can be expected that the inhibitory effect of *F. deltoidea* on alveolar bone resorption could be due to scavenging of the ROS during oxidative stress. It has been previously reported that antioxidants prevent and/or reduce the inflammatory state and bone resorption by inhibiting osteocyte apoptosis and reducing osteoclast activity, which in turn increases osteoblast activity and induces osteogenesis [32]. Furthermore, it also works by upregulating bone morphogenic protein (BMP) signaling pathways that promote osteoblast function and bone formation by reducing the effects of oxidative stress or chronic low-grade inflammation [13, 33–35].

In this study, the histological analysis revealed that the highest number of osteoclasts was present in the untreated ovariectomized group, while the estrogen-treated group had the lowest number of osteoclasts. In a previous study investigating the quantitative changes of osteoclast synthesis in the rat periodontium following ovariectomy, a significant increase in the number of osteoclasts attached to the bone in the ovariectomized group was observed as compared with the ovariectomized estrogen-treated group. Therefore, it was concluded that estrogen deficiency-induced osteoclastogenesis in the rat periodontium and the quantitative changes in osteoclastogenesis could be prevented by estrogen treatment [36].

The bone remodeling process involves the synthesis of bone matrix by osteoblasts and the coordinated resorption of bone by osteoclasts. The correlation observed between the increased number of osteoclasts and osteoporotic bone in the OVXN group is attributed to the disruption in the bone remodeling process due to the higher activity of osteoclasts as compared with osteoblasts, in which the rate of bone resorption becomes higher than bone synthesis, thereby resulting in osteoporosis [37].

The distinctive findings obtained in this study for bone resorption using the morphometric and histological analyses are due to the differences in the parameters observed for both analyses. For instance, although the detailed three-dimensional bone analysis is the main advantage of micro-CT, the bone morphometry representation in the analysis depends on the size of the cavity in the resorption area and its location in the bone [38]. In the presence study, micro-CT with a resolution of 9.0 *μ*m/pixel was operated to perform the bone morphometric analyses. It has been reported that an image resolution of 6.0 *μ*m/pixel or larger has limitation in viewing and analyzing bone resorption cavities accurately [39]. The ability to measure resorption cavities in three-dimensional images is likely to rely on image resolution, with cavities becoming more difficult to analyze in coarser images. High-resolution micro-computed tomography (synchrotron or nano-CT) can achieve a voxel size of 1 *μ*m or less and are capable of detecting resorption cavities [40, 41]. It was proposed that experimental studies of resorption cavities in three-dimensional images of the cancellous bone require images to be obtained at a resolution of 1.4 *μ*m/pixel in-plane or better to ensure accurate detection of resorption cavities. [39]. Hence, the limitations and nonstatistically significant findings of bone morphometric assessment in this study can be improved using high-image-resolution micro-computed tomography with a minimum of 1.4 *μ*m/pixel resolution.

In the micro-CT analysis, the representation of bone resorption was averaged and limited to simplified shapes, and hence the effect of bone resorption appears to be much more substantial than expected from the minimal bone loss [24]. On the other hand, in the H&E histological analysis, the two dimensions of bone resorption and formation areas are evaluated by quantifying the osteoclasts and osteoblasts in the region of interest. At the microstructural level, osteoclasts are responsible for creating bone loss by eroding the resorption cavities. Hence, the micro-CT analysis performed in this study did not fully represent the actual activities of bone resorption and bone formation within the study period. In contrast, the H&E histological analysis was able to evaluate the activity of bone resorption and formation by quantifying the osteoclasts and osteoblasts at the trabecular bone remodeling areas. Therefore, it is likely that the significant findings regarding the bone resorption in this study were due to the ability of the histological examination to evaluate and identify the initial activity of bone resorption following the bone remodeling process as opposed to the micro-CT analysis that measured bone quality based on their mineral properties and density [24].

## 5. Conclusions

Within the limitations of this study, bone morphometric analysis revealed that *F. deltoidea* did not exhibit significant effects on alveolar bone morphometry despite it showed to maintain the trabecular thickness and reduce trabecular separation in osteoporosis-induced Wistar rats. However, it has an antiresorptive effect as evidenced by its ability to reduce the number of osteoclasts and increase the number of osteoblasts in osteoporosis-induced Wistar rats, as indicated by the histological analysis. More studies are warranted in future, to illustrate the potential and mechanisms of *F. deltoidea* in preventing alveolar bone loss in osteoporosis-induced rats.

## Data Availability

The data used to support the findings of this study are available from the corresponding author upon request.
